# A preliminary ex vivo diffusion tensor imaging study of distinct aortic morphologies

**DOI:** 10.1111/joa.14223

**Published:** 2025-01-26

**Authors:** B. Tornifoglio, S. T. Robinson, R. E. Levey, A. J. Stone, S. Campisi, C. Kerskens, G. P. Duffy, S. Avril, C. Lally

**Affiliations:** ^1^ Trinity Centre for Biomedical Engineering Trinity Biomedical Sciences Institute, Trinity College Dublin Dublin Ireland; ^2^ Discipline of Mechanical, Manufacturing, and Biomedical Engineering School of Engineering, Trinity College Dublin Dublin Ireland; ^3^ Division of Vascular Surgery and Endovascular Therapy, Department of Surgery University of Florida College of Medicine Gainesville Florida USA; ^4^ North Florida/South Georgia Veterans Health System Gainesville Florida USA; ^5^ Disciple of Anatomy and Regenerative Medicine Institute, School of Medicine, College of Medicine, Nursing and Health Sciences, National University of Ireland Galway Galway Ireland; ^6^ Department of Medical Physics and Clinical Engineering St. Vincent's University Hospital Dublin Ireland; ^7^ Department of Cardiovascular Surgery University Hospital of Saint‐Etienne Saint‐Etienne France; ^8^ Trinity College Institute of Neuroscience, Trinity College Dublin Dublin Ireland; ^9^ Advanced Materials and Bioengineering Research Centre (AMBER) Royal College of Surgeons in Ireland and Trinity College Dublin Dublin Ireland; ^10^ Mines Saint‐Etienne Université Jean Monnet Saint‐Etienne, INSERM Saint‐Etienne France

**Keywords:** aneurysm, aortic disease, diffusion tensor imaging, dissection, magnetic resonance imaging

## Abstract

Changes in the microstructure of the aortic wall precede the progression of various aortic pathologies, including aneurysms and dissection. Current clinical decisions with regards to surgical planning and/or radiological intervention are guided by geometric features, such as aortic diameter, since clinical imaging lacks tissue microstructural information. The aim of this proof‐of‐concept work is to investigate a non‐invasive imaging method, diffusion tensor imaging (DTI), in ex vivo aortic tissue to gain insights into the microstructure. This study examines healthy, aneurysm and a type B chronic dissection aortae, via DTI. DTI‐derived metrics, such as the fractional anisotropy, mean diffusivity, helical angle and tractography, were examined in each morphology. The results from this work highlighted distinct differences in fractional anisotropy (healthy, 0.24 ± 0.008; aneurysmal, 0.19 ± 0.002; dissected, 0.13 ± 0.006) and a larger variation in the helical angle in the dissected aorta compared to healthy (39.28 ± 11.93° vs. 26.12 ± 4.60°, respectively). These differences were validated by histological characterisation. This study demonstrates the sensitivity of DTI to pathological changes in aortic tissue, highlighting the potential of this methodology to provide improved clinical insight.

## Graphical abstract

DTI‐derived tractography of distinct aortic pathologies.

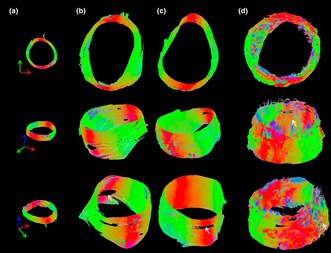

## 1 INTRODUCTION

The aorta is the primary conduit carrying blood directly from the heart to all major arterial tributaries, and as such requires adequate elasticity and compliance to accommodate the biomechanical forces resulting from physiologic blood flow of the cardiac cycle. Changes to the aortic wall microstructure can lead to luminal dilation (aneurysm) or intimal disruption (aortic dissection) resulting in aortic pathologies such as thoracic aortic aneurysm (TAA), or type B aortic dissection (TBAD). The natural history of TAA involves gradual aortic dilation leading to dissection or eventual rupture. Untreated ruptured TAA is universally lethal, so repair is recommended for both acute rupture and prophylactically when the theoretical rupture risk exceeds the risk of surgery (Upchurch Jr. et al., 2021). Dissection, and rupture, risk is currently assessed by measurement of maximum aortic diameter, but there is limited data to support the natural progression of untreated TAA (Cambria et al., 1995; Crawford & DeNatale, 1986; Griepp et al., 1999). Management of TBAD is dictated by both acuity and presence of features that categorise it as complicated (e.g. rupture, malperfusion of side branches) or uncomplicated (Lombardi et al., 2020). Initial management of acute, uncomplicated TBAD typically includes medical therapy with antihypertensive agents while surgical management is reserved for individuals at high risk of death or morbidity from dissection (MacGillivray et al., 2022). However, there is a growing body of literature that indicate early endovascular treatment in uncomplicated TBAD may improve aortic remodelling and long‐term survival (Iannuzzi et al., 2018; Qin et al., 2016).

Most descending thoracic aortic pathologies deemed to require intervention are preferentially treated by thoracic endovascular aortic aneurysm repair (TEVAR). TEVAR typically involves exclusion of the diseased segment of aorta with an endograft delivered minimally invasively. Advances in endograft technology and surgical technique have led to shorter length of stay and a decrease in perioperative mortality compared to open surgery (Bavaria et al., 2007; Chiu et al., 2019). However, optimal surgical planning is essential to avoid complications after TEVAR. Specifically, the increased endograft coverage length has been linked with increased risk of spinal cord ischemia (Martin et al., 2009; Scali et al., 2020). Additionally, degeneration of the proximal or distal aorta leads to up to 26% of patients requiring secondary interventions to support the initial aortic repair (Giles et al., 2019). Therefore, it is imperative to appropriately identify aortic pathologies at risk of rupture and optimise the length of the treated segment of aorta to reduce aortic coverage and risk of spinal cord ischemia, while limiting the incidence of secondary interventions.

Extensive research has gone into characterising the influence of specific microstructural components on the mechanics of aortic tissue (Chow et al., 2014; Duprey et al., 2010; Gundiah et al., 2013; Laffey et al., 2023; Mattson et al., 2017; Pal et al., 2014; Panpho et al., 2022; Pasta et al., 2016; Schriefl et al., 2015; Tsamis et al., 2013). Elastin distributes tensile stresses while collagen reinforces the vessel wall, together forming the load bearing components in the arterial wall (Chow et al., 2014; Duprey et al., 2016; Gundiah et al., 2013; Pasta et al., 2016). Degenerative processes in the presence of hypertension can lead to accelerated extracellular matrix degradation, apoptosis and elastolysis with hyalinisation of collagen (Akin, 2020). Ascending TAAs have been shown to have altered collagen orientation and significantly less elastin than healthy aortic tissue, which has direct mechanical implications on the tissue. Duprey et al. (2016) showed that the quality of collagen and elastin networks in the aortic wall contribute to tissue extensibility and strength. Panpho et al. found that the dissection flap (septum) (i) was stiffer than the true and false lumen wall, (ii) had the lowest collagen concentration, and (iii) lowest collagen to elastin ratio (Panpho et al., 2022). Given the connection between aortic pathologies and quantity, quality and alignment of microstructural components, herein lies an opportunity to seek methods to non‐invasively inform on these key changes.

In vivo magnetic resonance imaging (MRI) to investigate the integrity of the aortic wall is limited to a handful of studies (Botnar et al., 2014; Di Cesare et al., 2016; Richards et al., 2011; Takahashi et al., 2021). Inflammation (Richards et al., 2011) and turbulent flow in the false lumen (Takahashi et al., 2021) have been investigated in patients with aneurysm and dissection, respectively. In mice, Botnar et al. used an elastin‐specific MR imaging agent to obtain high spatial resolution of regions with ruptured elastic laminae (Botnar et al., 2014). A number of ex vivo MRI studies have investigated porcine arterial tissue with an aim to characterise the microstructural environment; specifically using diffusion tensor imaging (DTI) (Flamini et al., 2010; Flamini et al., 2013; Flamini et al., 2015; Shahid et al., 2017; Shahid et al., 2021; Tornifoglio et al., 2020; Wang et al., 2024). These studies not only identify the capability of DTI to non‐invasively determine the microstructure of arterial tissue but highlight the sensitivity to specific components—such as elastin (Tornifoglio et al., 2022) and smooth muscle cells (Shahid et al., 2021; Tornifoglio et al., 2020). Using superparamagnetic iron oxide particles, macrophages have been localised in excised abdominal aortic aneurysms (Umetsu et al., 2020). In particular, one recent study by Wang et al. investigated porcine tissue models and human ascending aortae ex vivo and found that DTI is capable of detecting medial degeneration, in particular that driven by changes in glycosaminoglycans (Wang et al., 2024). Chemical exchange saturation transfer has similarly been shown ex vivo to correlate to glycosaminoglycan content (Mortuza et al., 2022).

In this study we investigate three different thoracic aortic morphologies, healthy descending aorta, TAA from an ascending aorta, and, for the first time, a segment of chronic TBAD from the descending thoracic aorta. The aim of this study is to investigate if insight from diffusion tensor imaging has the potential to inform on aortic disease microstructures. Assessing this potential could ultimately move to improve current clinical indicators for rupture‐risk and guide the timing and extent of treatment.

## 2 METHODS

### 2.1 Human aortic specimens

Three different aortic morphologies were investigated in this study. Two specimens were obtained through the University of Galway Department of Anatomy. A healthy descending thoracic aortic segment was obtained from a 53‐year‐old male donor who died from a glioblastoma multiforme. A segment of descending thoracic aorta was obtained from an 83‐year‐old male who died from pneumonia, congestive cardiac failure and atrial fibrillation and who was found to have TBAD discovered incidentally during autopsy. All cadaveric material was bequeathed to the Medical School, University of Galway, for further advancement of medical knowledge. This is covered by the Medical Practictioners Act  in the Republic of Ireland. The whole human cadavers had been fixed with embalming fluid containing 21% methanol, 21% glycerine, 5.6% phenol and 3.1% formaldehyde. Tissue samples were dissected from whole donor cadavers by the study authors and stored in 70% ethanol before being transferred to phosphate‐buffered saline for storage prior to imaging.

Separately, two TAA specimens from the ascending aorta were harvested from patients undergoing elective surgery to replace their pathological segment of aorta with a synthetic graft. The first patient was a 70‐year‐old female with a bicuspid aortic valve, Sievers type I, the maximum diameter of her TAA was 53 mm. The second patient was a 69‐year‐old male with a bicuspid aortic valve, Sievers type I, the maximum diameter of his TAA was 54 mm. The collection was carried out with informed consent from patients and in accordance with the guidelines of the Institutional Review Board of the University Hospital Center of Saint‐Etienne. These two surgical specimens were also fixed, with 4% formaldehyde buffered solution (pH 6.9), prior to MR imaging.

### 2.2 MR imaging

All samples were imaged individually in a small‐bore (30 cm) horizontal 7 Tesla Bruker BioSpec 70/30 USR system (Bruker, Ettlinger, Germany). Specimens were secured to a 3D printed holder which was secured in a horizontal tube with fresh phosphate buffered saline for imaging and the lumen of each specimen was parallel with the magnetic field. A conventional 2D spin echo DTI sequence was acquired, where *b*‐values = 0, 800 s/mm^2^ with 32 diffusion directions. A 2D spin echo DTI sequence was used for all four samples. The healthy aorta was imaged with the following parameters: TE/TR: 18.182/1500 ms, 5 averages, 5 slices, image size: 128 × 128, field of view: 64 mm × 64 mm, *b*‐values: 0, 800 s/mm^2^, 64 diffusion directions, gradient duration 3.8 ms, gradient separation: 8.802 ms and acquisition time: 17 h and 20 min. The TBAD aorta had the same parameters save TR: 1182.335 ms, 55 slices and acquisition time of 13 h and 39 min. Both TAA samples were imaged with the following parameters: TE/TR: 18.182/4000 ms, 4 averages, 20 slices, image size: 140 × 140, field of view: 70 mm × 70 mm, fat suppression on, *b*‐values: 0, 800 s/mm^2^, 32 diffusion directions, gradient duration 3.8 ms, gradient separation: 8.802 ms and acquisition time: 20 h and 32 min. Contrast weighting between the sequences were compared and negligible differences found (Elster, 1988).

### 2.3 MR data processing

Pre‐processing of the raw data and fitting of the diffusion tensor follow previously established pipelines (Kellner et al., 2016; Leemans et al., 2009; Tornifoglio et al., 2020; Tournier et al., 2019; Veraart et al., 2016). Prior to any data processing, the number of diffusion directions for the healthy and chronically dissected aorta were down sampled to 32 directions in DSI Studio (http://dsi‐studio.labsolver.org) in order to match the aneurysmal aorta acquisition. All raw data was denoised (Veraart et al., 2016) and corrected for Gibbs ringing (Kellner et al., 2016) in MRtrix3 (Tournier et al., 2019) (http://mrtrix3.org) prior to fitting the mono‐exponential tensor model in ExploreDTI (Leemans et al., 2009). Two scalars were calculated on a voxel‐wise basis from the tensor, allowing for parametric maps of each sample. Fractional anisotropy (FA) is a scalar representation of the degree of anisotropic diffusion, where a higher value corresponds to more anisotropic diffusion, indicative of more underlying microstructural organisation. Mean diffusivity (MD) is the average diffusion occurring in all directions within a voxel; increased MD corresponds to more diffusion occurring. Helical angles (HA) were calculated from the first eigenvector and represent the angle between the first eigenvector (principal direction of diffusion) and the plane normal to the main magnet field (transverse slice of aortic segment), as done previously in ex vivo arterial tissue (Tornifoglio et al., 2023).

Tissue masks were generated to probe these DTI metrics within the tissue. To avoid partial volume effects, a previously used masking methodology was used (Tornifoglio et al., 2022). Briefly, low signal regions were removed as were regions with high residuals from the tensor fitting, and then the background medium (phosphate buffered saline). Deterministic tractography was performed via ExploreDTI in order to visualise the underlying microstructural alignment, or lack thereof, of the three morphologies. Seed points matched the resolution (0.5 × 0.5 × 0.5 mm) and the FA threshold and tracking range was 0.05 and 0.05–1, respectively. Tract length was prescribed to be between 15 and 50 mm based on the size of the samples. The angular threshold was 45° and the step size 0.5.

### 2.4 Histological processing

Standard histological analysis was performed on tissue sections from each aorta. The sections were stained with Masson's Trichrome with Gomori's aldehyde fuchsin staining. Aneurysmal samples were stained with haematoxylin & eosin, Verhoeff's elastin and picrosirius red. Brightfield images are presented for all stains, with polarised light microscopy presented for the picrosirius red stained sectioned as well.

### 2.5 Regions of interest

All aortic samples were compared to each other without differentiating any specific regions of interest (ROIs). Specifically, the mean DTI metric per sample was compared. However, ROIs within the TBAD sample were identified by an experienced physician (S.T.R). Four regions were identified – media, dissected media, septum and luminal thrombus. The septum is the dissection flap which covers the false lumen, while the dissected media is behind the false lumen and luminal thrombus.

### 2.6 Statistical methods

Quantitative MR data is presented in violin plots with the mean for each slice shown by individual points; however to avoid pseudo replication (Lazic, 2010), no statistical analysis is presented.

## 3 RESULTS

### 3.1 Histologic analysis of aortic tissue

**FIGURE 1:**
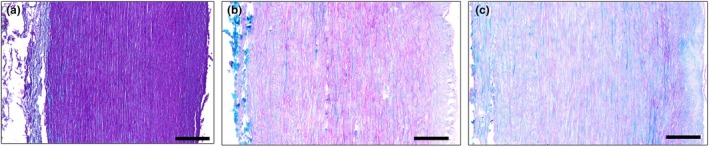
Example Masson's Trichrome stained cross‐sections of the (a) healthy and (b, c) thoracic aortic aneurysmal segments. Scale bars are 200 μm.

The healthy aorta and TAA specimens had a uniform medial layer with luminal intima and outer adventitia (Figure 1).

**FIGURE 2:**
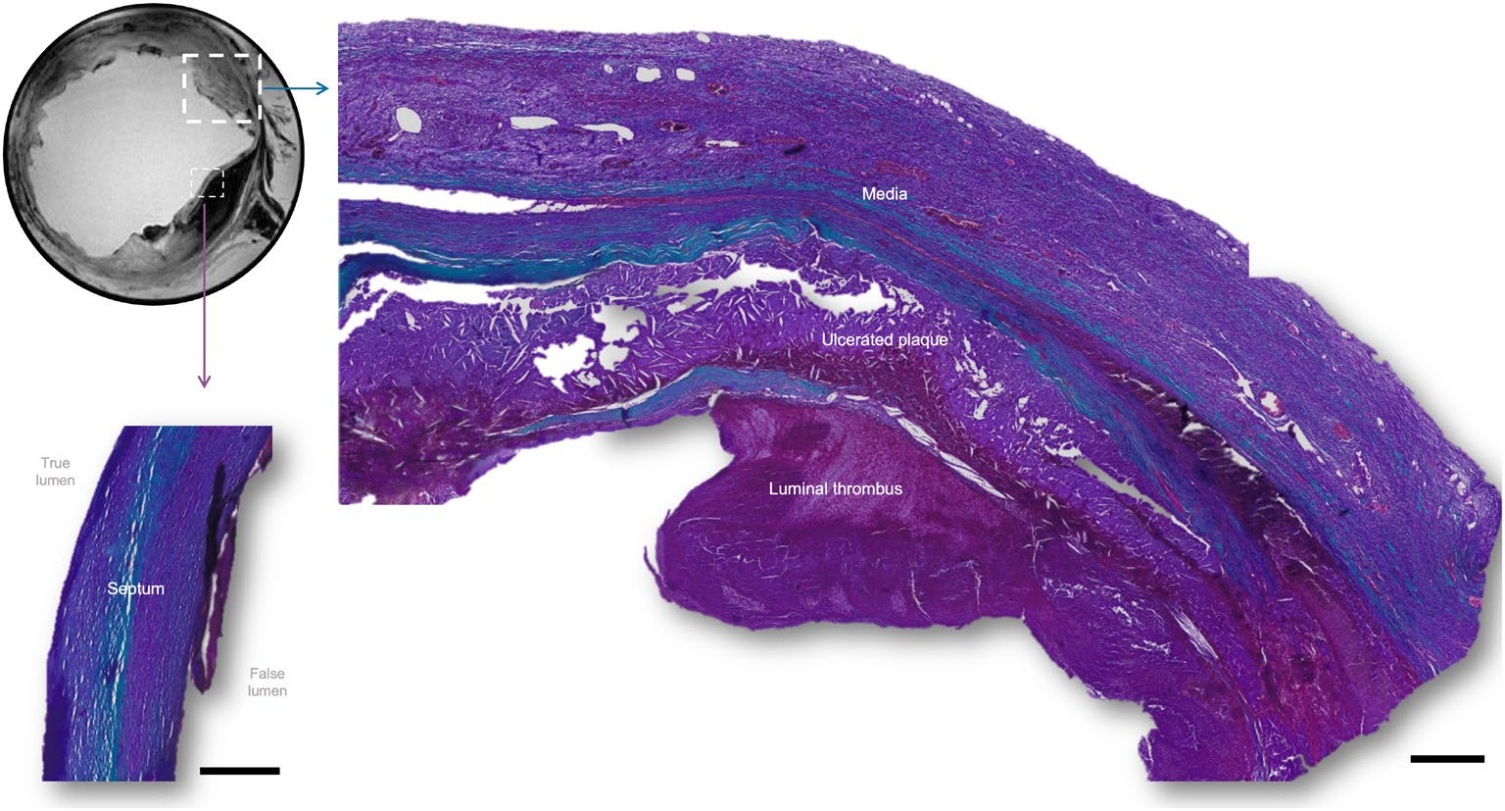
Example Masson's Trichrome stained cross‐section of the complex diseased descending thoracic aortic segments. T1‐weighted MRI image highlights where the histological images are representing. The true lumen and false lumen locations are highlighted in grey text; however the false lumen is not visualised here. Scale bars are 500 μm.

The TBAD is a complex lesion consisting of a completely thrombosed false lumen (not shown) with thickened septum (Figure 2). There was also an ulcerated plaque, present with cholesterol crystals, and luminal thrombus. Additional histological images of the TBAD are included in Supplementary material.

**FIGURE 3:**
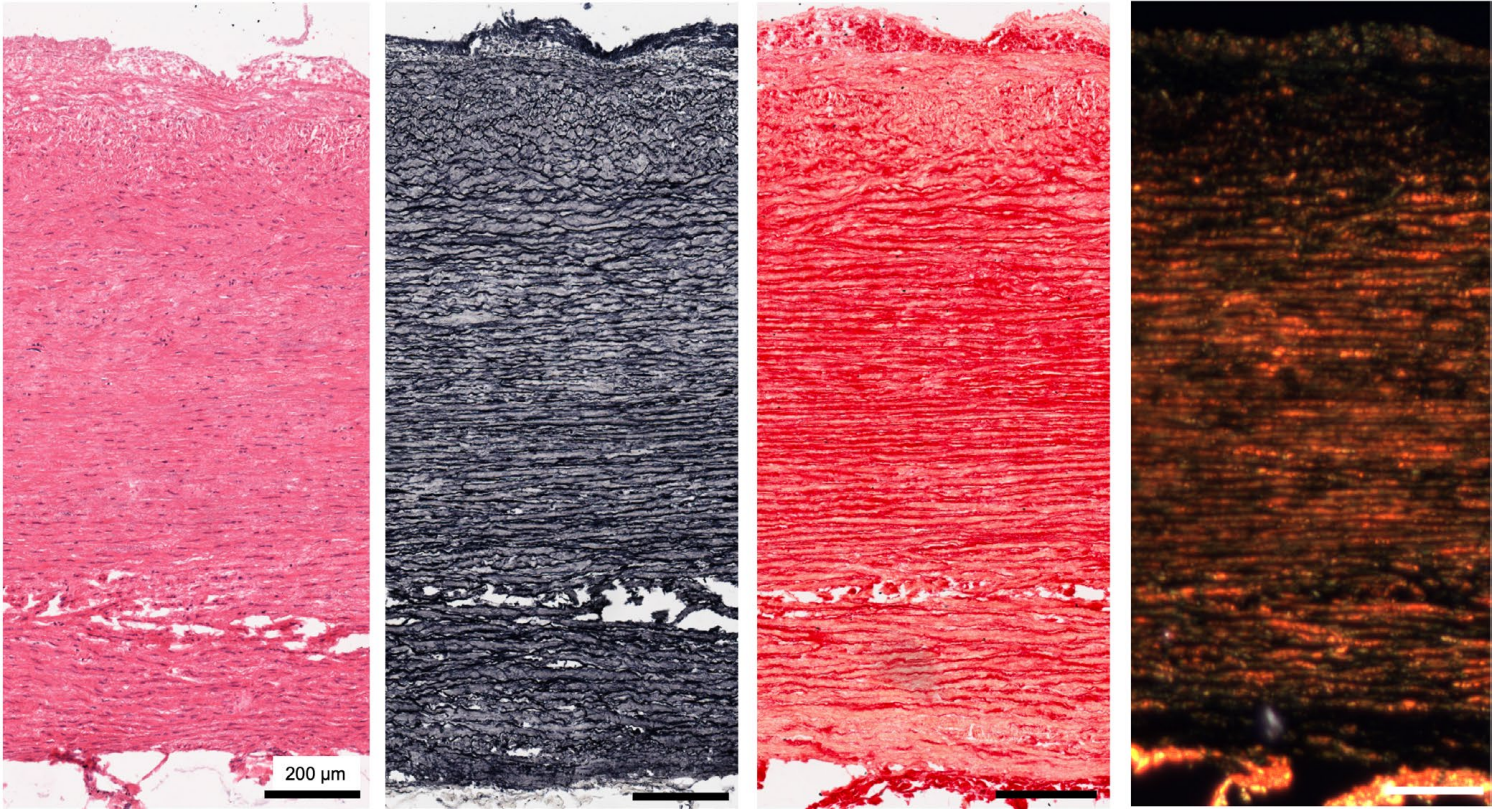
H&E, Verhoeff's elastin and picrosirius red (brightfield and polarised light microscopy) images of TAA sample 1.

**FIGURE 4:**
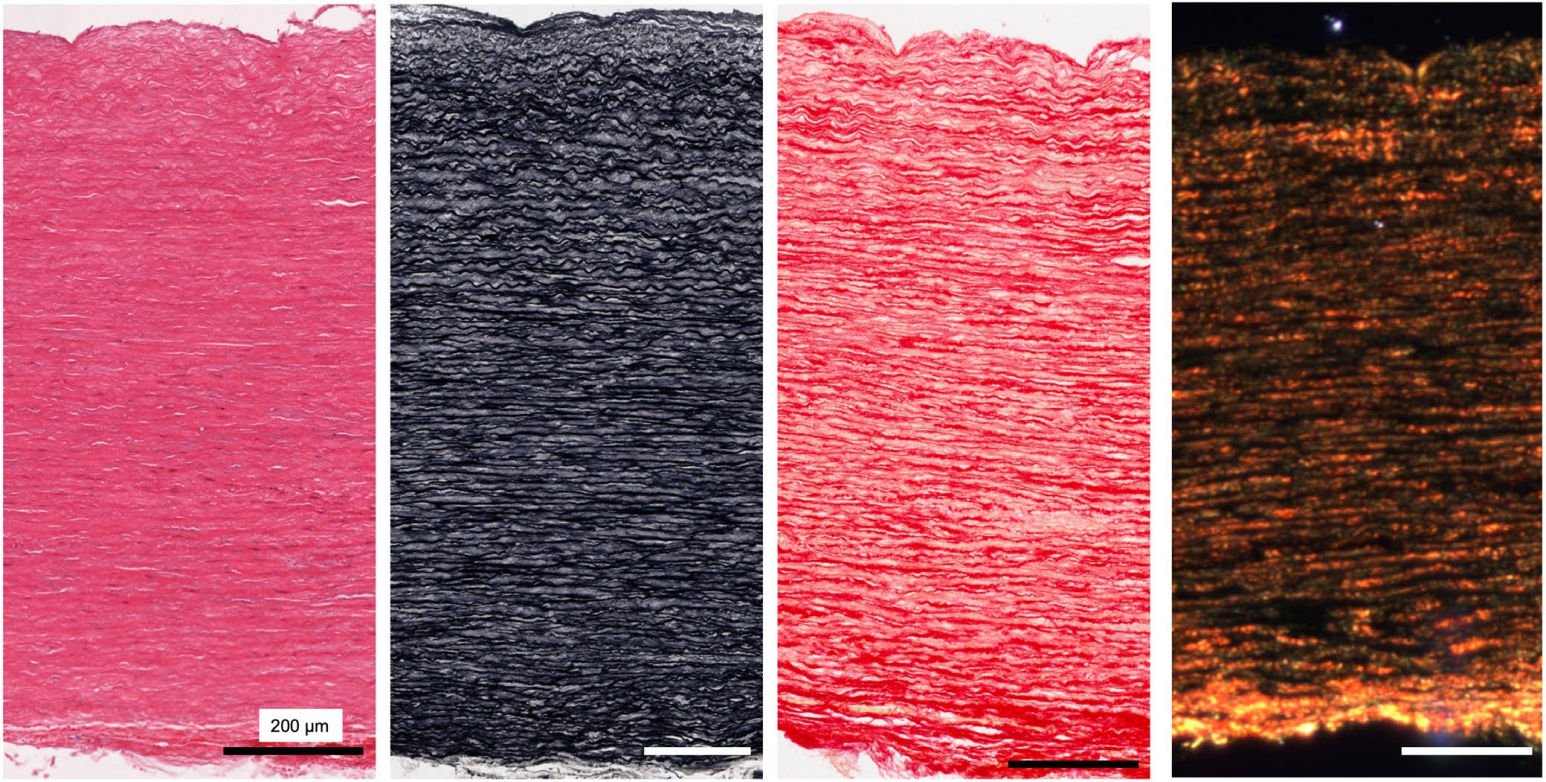
H&E, Verhoeff's elastin and picrosirius red (brightfield and polarised light microscopy) images of TAA sample 2.

A low cell density in the aneurysmal samples can be observed in H&E stained sections of the TAA samples; however, with both elastin and collagen present and aligned (Figures 3 and 4).

### 3.2 DTI metrics across three different aortic morphologies

**FIGURE 5:**
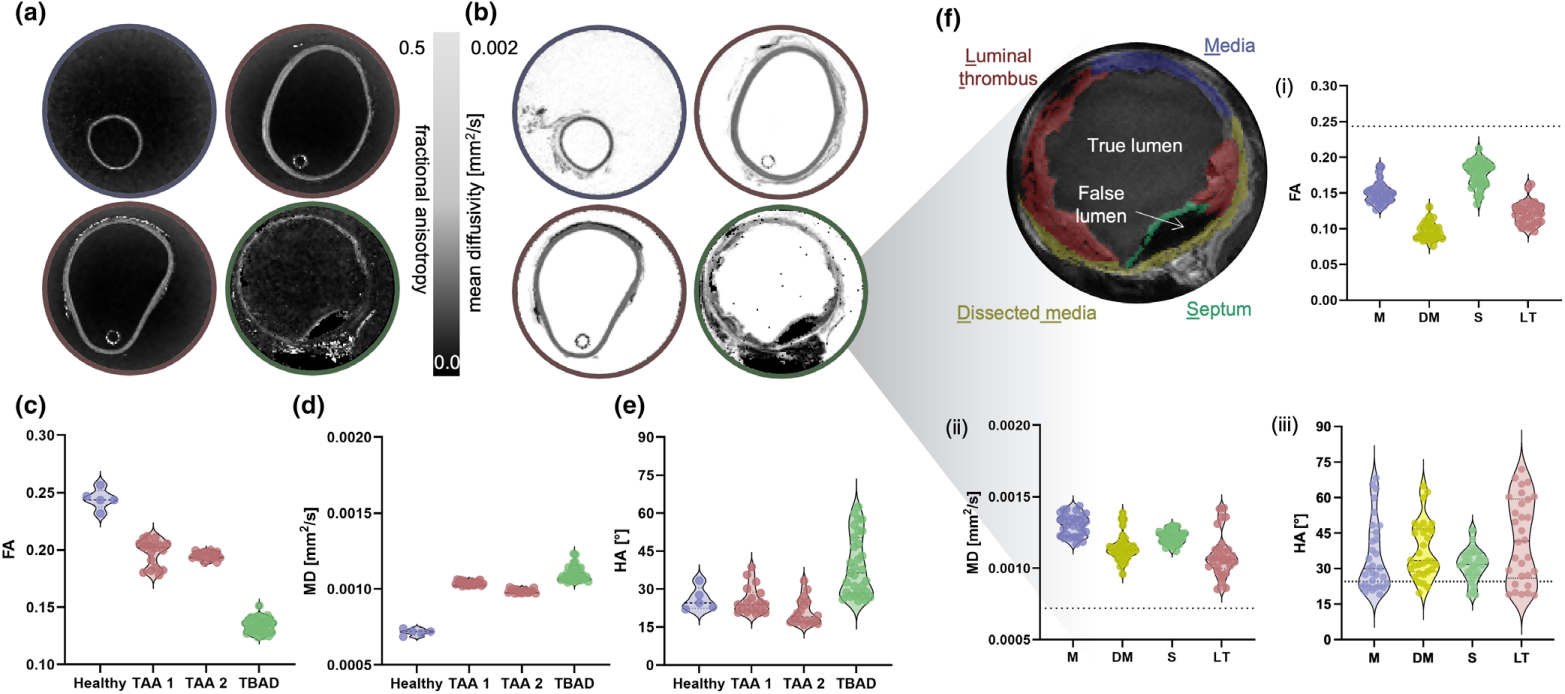
DTI metrics across three separate aortic morphologies. Parametric maps of (a) fractional anisotropy (FA) and (b) mean diffusivity (MD). (c–e) Violin plots of (c) FA, (b) MD, and (c) helical angle (HA). Dashed lines within the violin are the median and each data point is the mean metric per MR slice; *n* = 5 slices for healthy, *n* = 18 slices for TAA1 and TAA2, and *n* = 31 slices for TBAD. (f) Distinct regions within a chronic dissected aorta were looked at – the media (M, blue), dissected media (DM, yellow), septum (S, green) and luminal thrombus (LT, in red). (f, i–iii) Violin plots of the DTI‐derived metrics for each slice within each region. The dashed line in each plot is the average metric for healthy aorta.

Parametric maps of FA and MD and the mean FA and MD per slice for each aortic segment were calculated (Figure 5). Healthy aortic tissue had the highest degree of anisotropic diffusion (0.24 ± 0.008) and the TBAD sample the lowest (0.13 ± 0.006), which was noticeably lower than all samples. Conversely, the healthy aorta had the lowest MD of all samples (7.2 ± 0.18 × 10^−4^ mm^2^/s). The HA for each specimen is presented in Figure 5(e), with the TBAD sample having the largest spread (39.28 ± 11.93°).

### 3.3 DTI metrics within a chronic dissected aorta

The presence of multiple different morphological features within the TBAD sample motivated further investigations into these distinct regions. Figure 5f shows four ROIs – media (M, blue), dissected media (DM, yellow), septum (S, green) and luminal thrombus (LT, red). The true and false lumens are also labelled. The DTI‐derived metrics in each of these ROIs are also presented in comparison to the mean value of healthy aorta (dashed line).

### 3.4 Tractography

**FIGURE 6:**
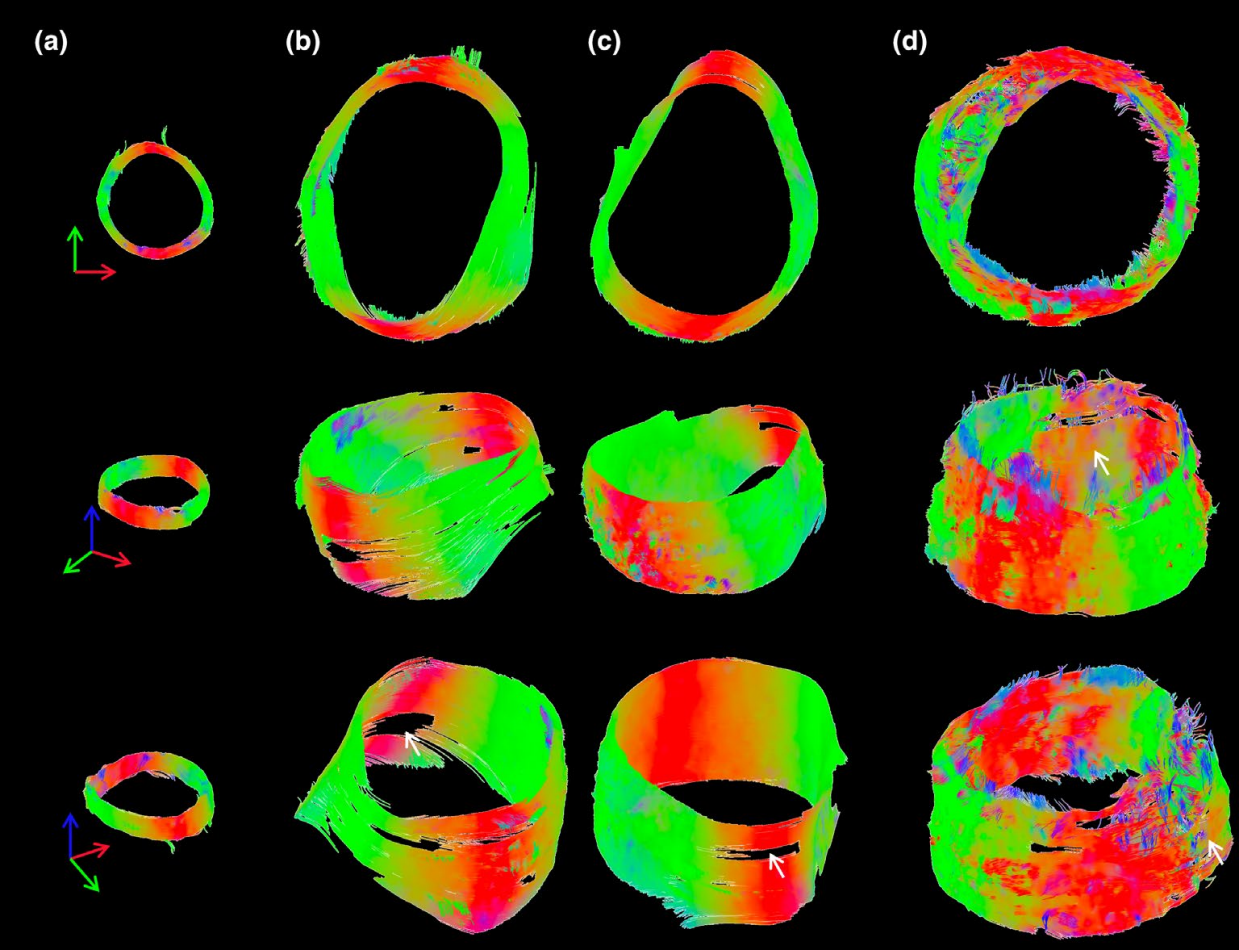
Tractography of aortic samples. Three views for the (a) healthy, (b, c) thoracic aortic aneurysmal and (d) type B dissected aortic samples. Red‐green tractography corresponds to circumferential orientation while blue represents more axial. White arrows in point to areas of less densely packed tracts in the aneurysmal samples (b, c) and to the septum (middle row) and false lumen (bottom row) in the TBAD sample (d).

Tractography was performed for each sample (Figure 6). The healthy aorta is characterised by continuous, circumferential alignment. When looking at the aneurysmal samples (b, c), while still predominantly circumferential, there are regions where the tracts are not continuous or are less densely packed (white arrows). More disorganisation is evident in the TBAD sample (d) overall. The septum can be visualised (white arrow) covering the false lumen thrombus, while there are very few tracts characterising the dissected media behind this false lumen (white arrow, bottom right).

## 4 DISCUSSION

The need for better clinical indicators of the stability and rupture‐risk of the aortic wall in aortic diseases is as prevalent as ever as the burden of aortic diseases increases (Bossone & Eagle, 2021; Hagan et al., 2000; Melvinsdottir et al., 2016; Sampson et al., 2014). Current guidelines for management and surveillance of aortic disease are frequently based on diameter thresholds that predicate rupture risk (Upchurch Jr. et al., 2021). For abdominal aortic aneurysms, rupture risk has been predicted based on several seminal autopsy studies (Darling et al., 1977; Estes, 1950; Schatz et al., 1962; Szilagyi et al., 1966), while more contemporary analyses have suggested rupture risk at specified maximum aortic diameters may actually be lower than that which has been historically reported (Rokosh et al., 2021). Diameter thresholds for repair of the thoracic aorta are also vague, with rupture risk established by single centre studies (Davies et al., 2002; Kim et al., 2015). The imaging technique presented here could be advantageous in surveillance screening of at risk cohorts, such as those with Marfan's syndrome (Ramirez et al., 2018). Rupture or dilation of the aorta occurs from biomechanical weakening of the wall and therefore, mechanically sensitive clinical indicators are advantageous. The influence of microstructure on the mechanics of the aortic wall is well established (Burton, 1954), furthering the motivation for non‐invasive microstructural insight into the wall. DTI has sensitivity to specific microstructural components in arterial tissue (Shahid et al., 2021; Tornifoglio et al., 2020; Tornifoglio et al., 2022; Wang et al., 2024) and the current study harnesses that sensitivity to assess distinct aortic pathologies.

### 4.1 Healthy aortic tissue

The DTI‐derived metrics utilised here allowed for a more quantitative characterisation of the aortic morphologies compared to conventional anatomical imaging. The arterial wall is a highly organised, anisotropic structure composed of elastin, collagen and smooth muscle cells (Gundiah et al., 2013; O'Connell et al., 2008). The calculated helical angles, indicative of the dominant angle of diffusion, of healthy aorta in this study agree very well with previously published results (Flamini et al., 2013; Flamini et al., 2015). Anisotropic diffusion has previously been measured in healthy porcine carotids (Shahid et al., 2021; Tornifoglio et al., 2020) and aorta (Wang et al., 2024). It has also been measured in human aged cadaveric carotids (mean age 78.25 ± 11.2 years) (Tornifoglio et al., 2022) and fresh ascending aortae (mean age 61.56 ± 13.3 years) (Wang et al., 2024). The FA values in healthy aorta presented in this study fall below healthy porcine tissue (Tornifoglio et al., 2020), are higher than the aged cadaveric common carotids (Tornifoglio et al., 2022) and in line with results from Wang et al. given the age (Wang et al., 2024). Vascular ageing affects both mechanical and structural properties of the arterial wall, specifically the loss of elastin leading to a reduction in wall compliance (Jani & Rajkumar, 2006; Sertedaki et al., 2020). While the novel work by Wang et al. includes specimens from a range of ages, future studies should look to decouple the observed decrease in FA due to vascular aging and the decrease in FA due to disease‐related changes.

### 4.2 Aneurysmal aortic tissue

FA has been shown to be highly correlated with elastin content—with decreasing elastin yielding decreasing measurable anisotropic diffusion (Tornifoglio et al., 2022; Wang et al., 2024). The FA in the aneurysmal samples was less than that in the healthy aorta and the MD notably higher. This suggests that both FA and MD might be useful metrics in surveillance imaging of aneurysmal tissue. Rather than relying solely on the aortic diameter these metrics could offer insight into progressive elastin fragmentation and depletion which would be evident from decreasing FA and increasing MD. The loss of elastin in arterial walls is often associated with a stiffness increase of the wall (Gundiah et al., 2013) and a release of intrinsic compressive stresses on the collagen fibres (Chow et al., 2014), allowing them to become disordered (Chow et al., 2013). Elastin fragmentation, fibrosis and glycosaminoglycan accumulation are believed to be adaptations to stress and trauma in the arterial wall (Peterss et al., 2016; Schlatmann & Becker, 1977); however, how these microstructural changes develop and progress over time has not been studied. The potential for non‐invasive metrics to characterise changes in the microstructure over time could offer novel insights which ultimately can be linked to mechanics. The spatial insight observed via tractography could also highlight vulnerable regions of aneurysmal tissue which could also help guide surgical planning for endografts.

### 4.3 DTI‐derived metrics in a chronic TBAD specimen

TBAD are a complex clinical entity, and there is ongoing debate over optimal treatment strategies. Complicated TBAD (e.g. with malperfusion or concerning radiographic features) is preferentially treated with TEVAR and coverage of the entry tear (Investigators, 2014; Zipfel et al., 2011). Uncomplicated TBAD are frequently treated medically and in time they transition from acute to chronic dissections (Durham et al., 2015) with 38% of patients requiring eventual intervention (Schwartz et al., 2018). Early surgical intervention is currently not recommended, but clinical trials indicate TEVAR is safe and may improve survival and prevent disease progression (Nienaber et al., 2013) in acute uncomplicated TBAD. There is therefore a need to identify patients that would benefit from early surgical intervention, and help guide optimal treatment strategies while minimising risks or complications of the procedure. The DTI‐derived metrics in the present study are sensitive to microstructural changes which occur in the septum and dissected media around the false lumen, where rupture would likely occur in TBAD.

Certain morphological characteristics have been shown to predict late aortic degeneration after TBAD (Tolenaar et al., 2013). A thrombosed false lumen, when compared to a patent false lumen, has a beneficial effect on aortic remodelling, whereas a false lumen with both flow and thrombosis is associated with increased mortality (Tsai et al., 2007). Septum stiffness is accepted as a marker for chronicity; however, this has not been systematically observed (Karmonik et al., 2012). Microstructurally, this stiffness could be due to increased elastin fragmentation present and accumulation of glycosaminoglycans, both of which occur with natural ageing and chronic dissection progression (Bode‐Janisch et al., 2012; Peterss et al., 2016; Schlatmann & Becker, 1977). Loss of elastin coupled with elastin fragmentation has been shown to cause biomechanical weakening in the false lumen (Panpho et al., 2022), while the septum shows stiffer behaviour compared to the false and true lumen and the lowest collagen to elastin ratio (Panpho et al., 2022). The septum (also referred to as the dissection flap) presented in the complex TBAD specimen in this study demonstrated an FA similar to that of the media of the true lumen. This suggests that the microstructure in this region maintains key microstructural components that drive measurable anisotropic diffusion—elastin, cells, and collagen. In the dissected media, located behind the false lumen, a drop in FA and increase in MD was observed. This highlights a compromised microstructure which lacks anisotropy and might be mechanically more susceptible to rupture (Tornifoglio et al., 2023). This was further visualised when looking at the tractography of the TBAD sample and confirmed histologically. The septum was visualised by more aligned tractography, whereas the dissected media yielded little to no alignment. It is worth noting the complex nature of this TBAD specimen—both atherosclerotic and dissected. The different microstructures present in this sample, for example the cholesterol clefts and thrombus, undoubtably impact the diffusion metrics in the tissue which would be an interesting investigation in future studies. In particular, diffusion weighted imaging has shown the ability to differentiate thrombus age and composition (Toussaint et al., 1997; Viereck et al., 2005).

### 4.4 Clinical relevance

The translation of utilising DTI for assessment of arterial tissue in vivo is complicated by scan restrictions including the need for high resolution, no motion, and extended scan time. The current study was feasible as it had an optimised ex vivo set‐up, allowing for a detailed investigation of aortic tissues without imaging time restrictions. Opriessnig et al. demonstrated, for the first time, the feasibility of using in vivo DTI to measure diffusion anisotropy in cross‐sections of the carotid arteries (Opriessnig et al., 2016). By utilising a read‐out segmented echo planar pulse sequence with a 2D acquisition, they were able to achieve clinically acceptable scan times and sufficient signal‐to‐noise for DTI data. They also used peripheral pulse triggering to combat pulsatile vessel motion and cardiac triggering to acquire images in the diastolic phase. Results from their study suggest that 2D diffusion measurements perpendicular to the artery's axis would be sufficient for obtaining DTI parameters, as the dominant diffusion direction occurred in‐plane circumferentially, as seen by the helical angle results. This would then make the imaging protocol feasible for clinical translation.

### 4.5 Limitations

A limitation of the current study is the small sample size of the pathologies investigated. To demonstrate feasibility of DTI of the aorta, we required intact whole tissues which were procured from available cadaveric specimens and from patients undergoing elective surgery. Thus sample size was limited and ultimately more samples are needed. Additionally, there is variability in our aortic specimens as a result of anatomic location, with the TAA specimens procured from the ascending aorta and the healthy and TBAD specimen derived from the descending aorta. Differences in fixation can also affect the diffusive properties within tissues and can differ from fresh tissue (Agger et al., 2015; Giannakidis et al., 2016; Mazumder et al., 2016), although in this study all specimens were prepared in a similar fashion. While similar, the fixatives used on the cadaveric specimens versus the surgical specimens are different, and for this reason histology between them could not be qualitatively compared. Despite these limitations, our results demonstrate an important proof of concept that orientation independent DTI parameters, FA and MD, can be used to characterise whole aortic tissue in a manner which can help guide clinical decision making. It is important to note that the segments in this study did not display significant curvature or tortuosity; however, in the case where there these geometrical features exist, it is likely the HA measurement would need to be considered based on the plane normal to the lumen rather than the magnetic field. Additionally, challenges around adequate resolution to identify and characterise small morphological features (<1 mm) in vivo will need to be overcome to avoid metric‐bias from low signal‐to‐noise (Farrell et al., 2007), but also avoid partial volume effects. While literature exists on diffusion models and reconstruction methods to handle fibre orientation dispersion in the brain (Schilling et al., 2018), this work remains to be done in diseased vascular tissue where crossing fibres may exist.

With full acknowledgement of the translational hurdles, the authors hope that the results from this study motivate further development of clinically feasible imaging sequences for the characterisation of aortic tissue, and more specifically, for identifying aortic pathologies.

## Supporting information


Data S1.

Data S2.

Data S3.

Data S4.

## Data Availability

All data is available from the corresponding author on reasonable request.
