# Supplementary figures and images for: A preliminary ex vivo diffusion tensor imaging study of distinct aortic morphologies
Source: J Anat. 2025 Jan 26;246(5):745–56. doi: 10.1111/joa.14223 (PMC11996718; doi:10.1111/joa.14223)

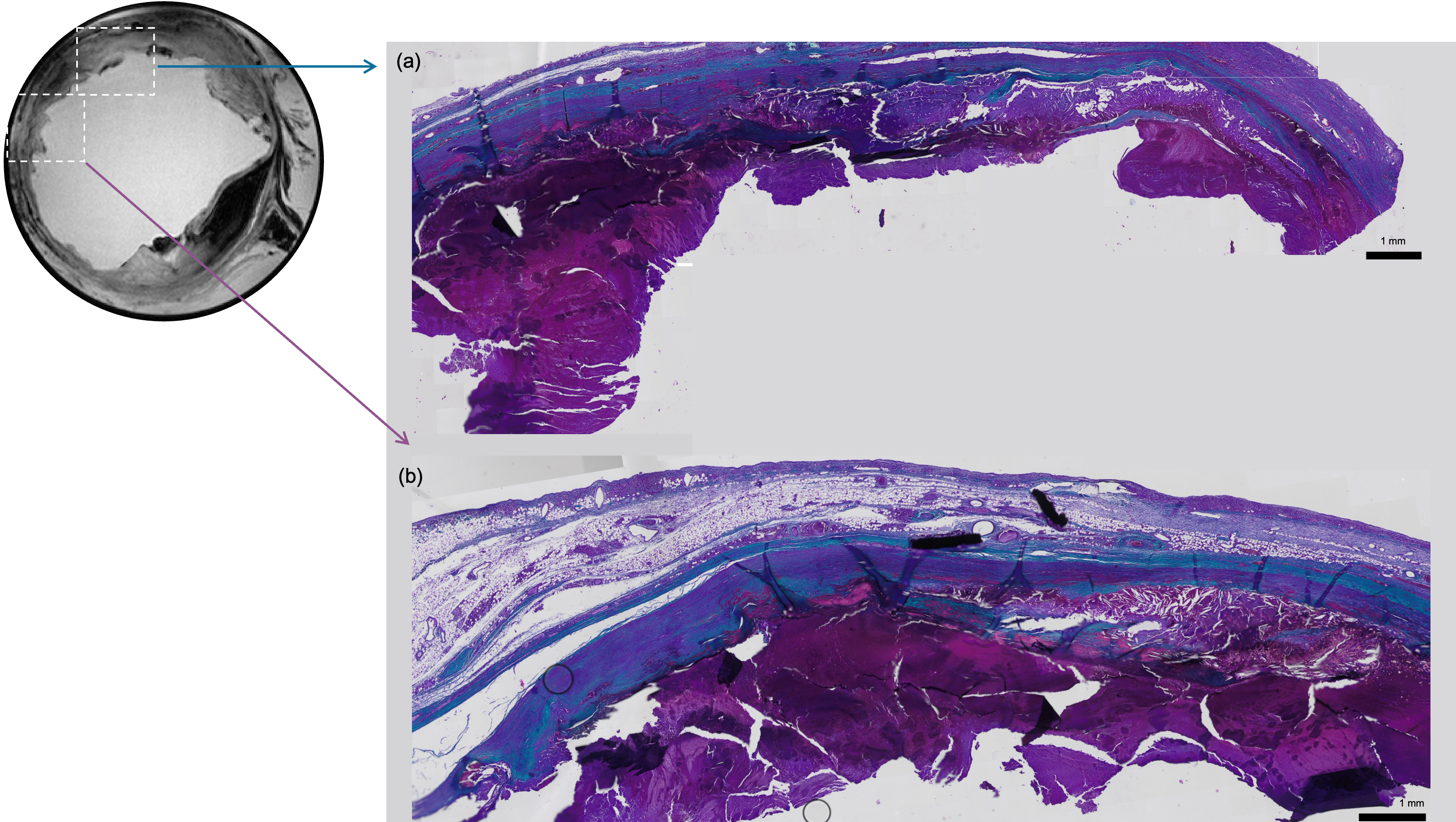

Supplement: Supplementary file 1 — Data S1. [file JOA-246-745-s003.tiff]

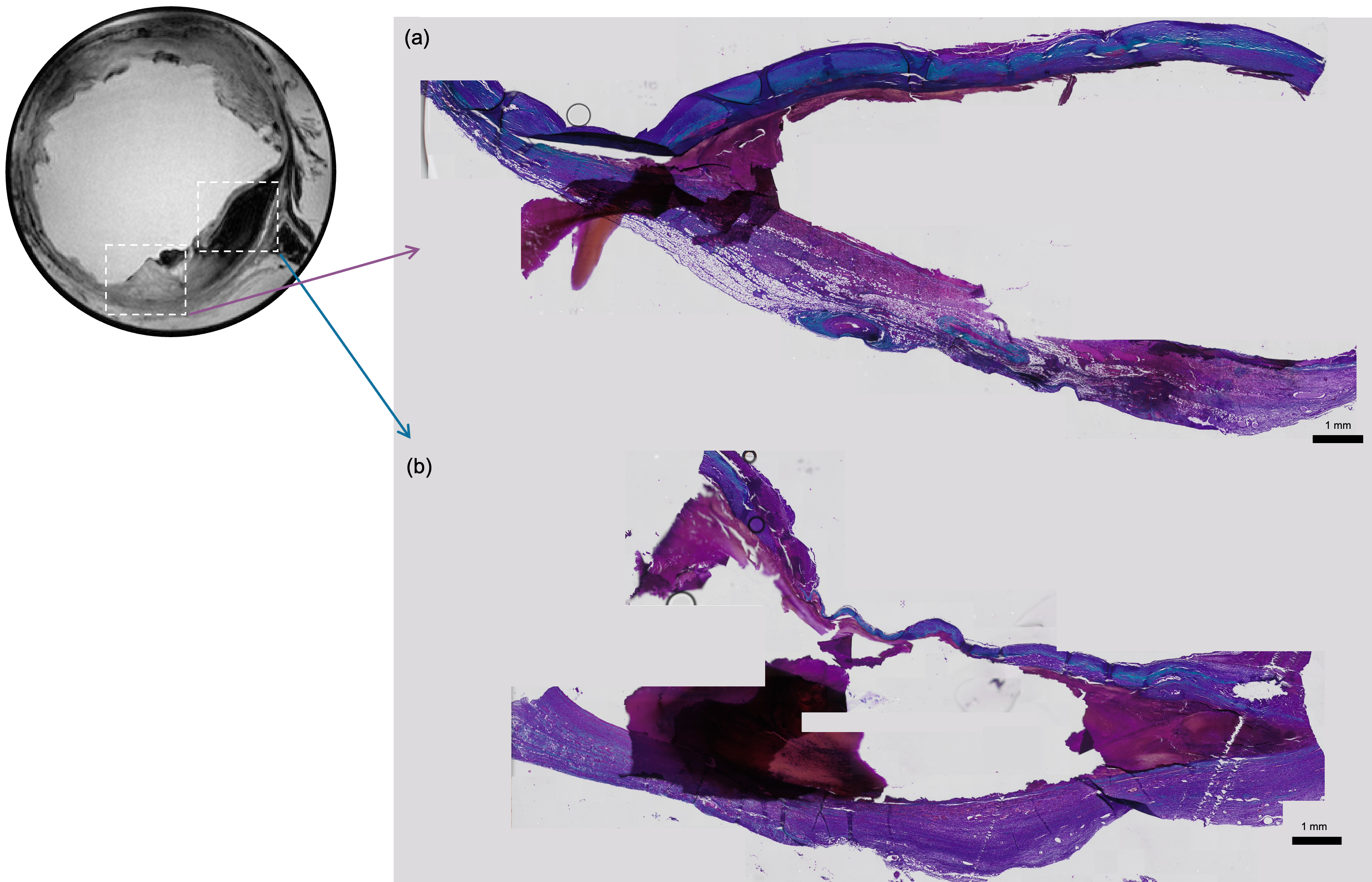

Supplement: Supplementary file 2 — Data S2. [file JOA-246-745-s004.tiff]

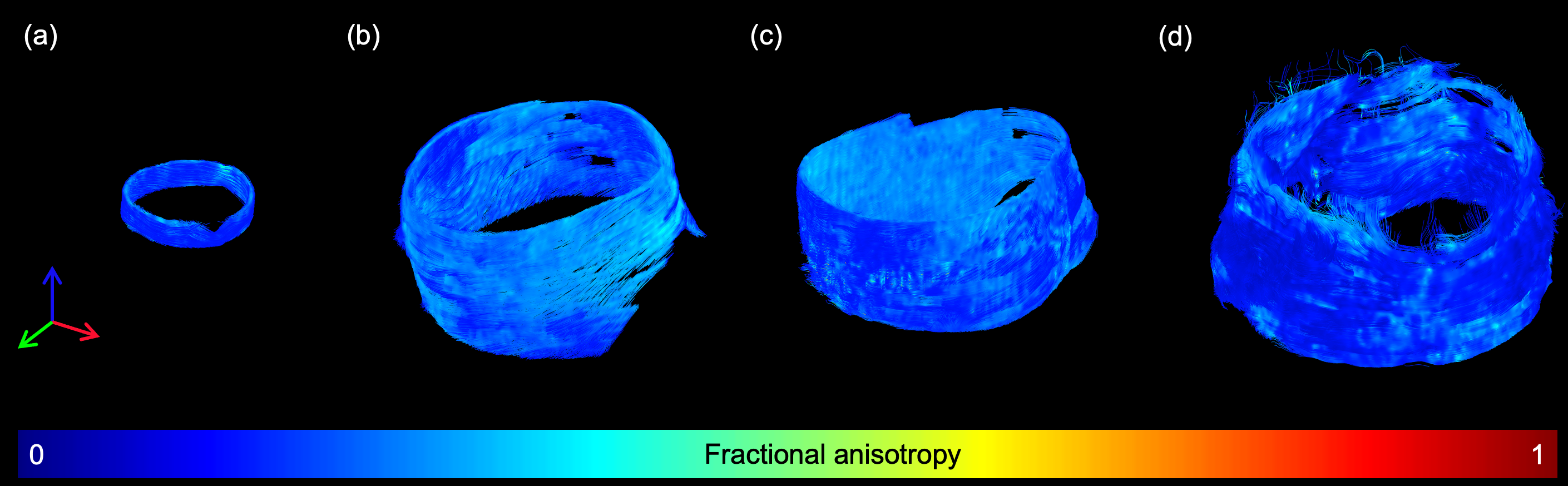

Supplement: Supplementary file 3 — Data S3. [file JOA-246-745-s002.tiff]
