# Supplementary material for: A preliminary ex vivo diffusion tensor imaging study of distinct aortic morphologies
Source: J Anat. 2025 Jan 26;246(5):745–56. doi: 10.1111/joa.14223 (PMC11996718; doi:10.1111/joa.14223)
Supplement: Supplementary file 4 — Data S4. [file JOA-246-745-s001.pdf]

A preliminary *ex vivo* diffusion tensor imaging study of distinct aortic morphologies

B. Tornifoglio, S.T. Robinson, R.E. Levey, A.J. Stone, S. Campisi, C. Kerskens, G.P. Duffy, S. Avril, C. Lally

Supplementary information

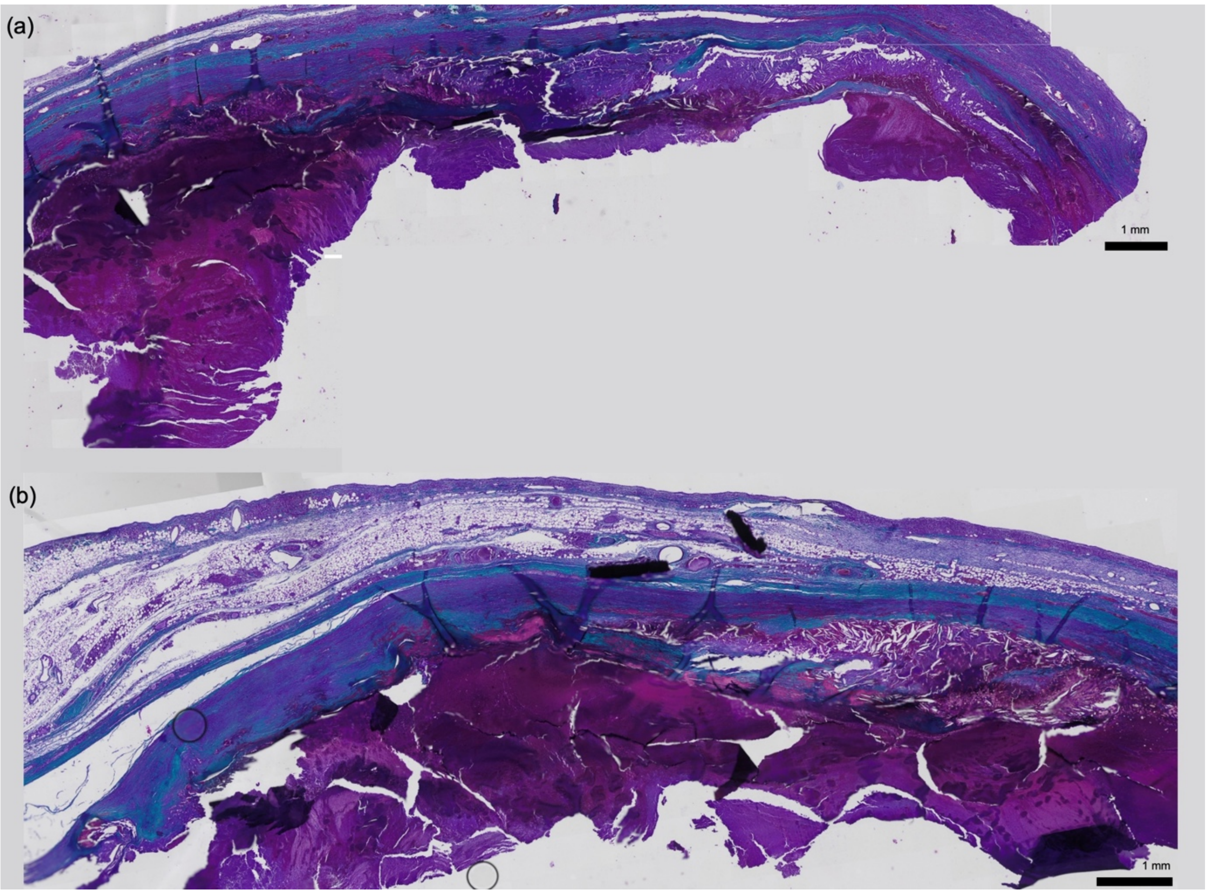

Supplementary figure 1. Additional example histological images of the TBAD specimen.

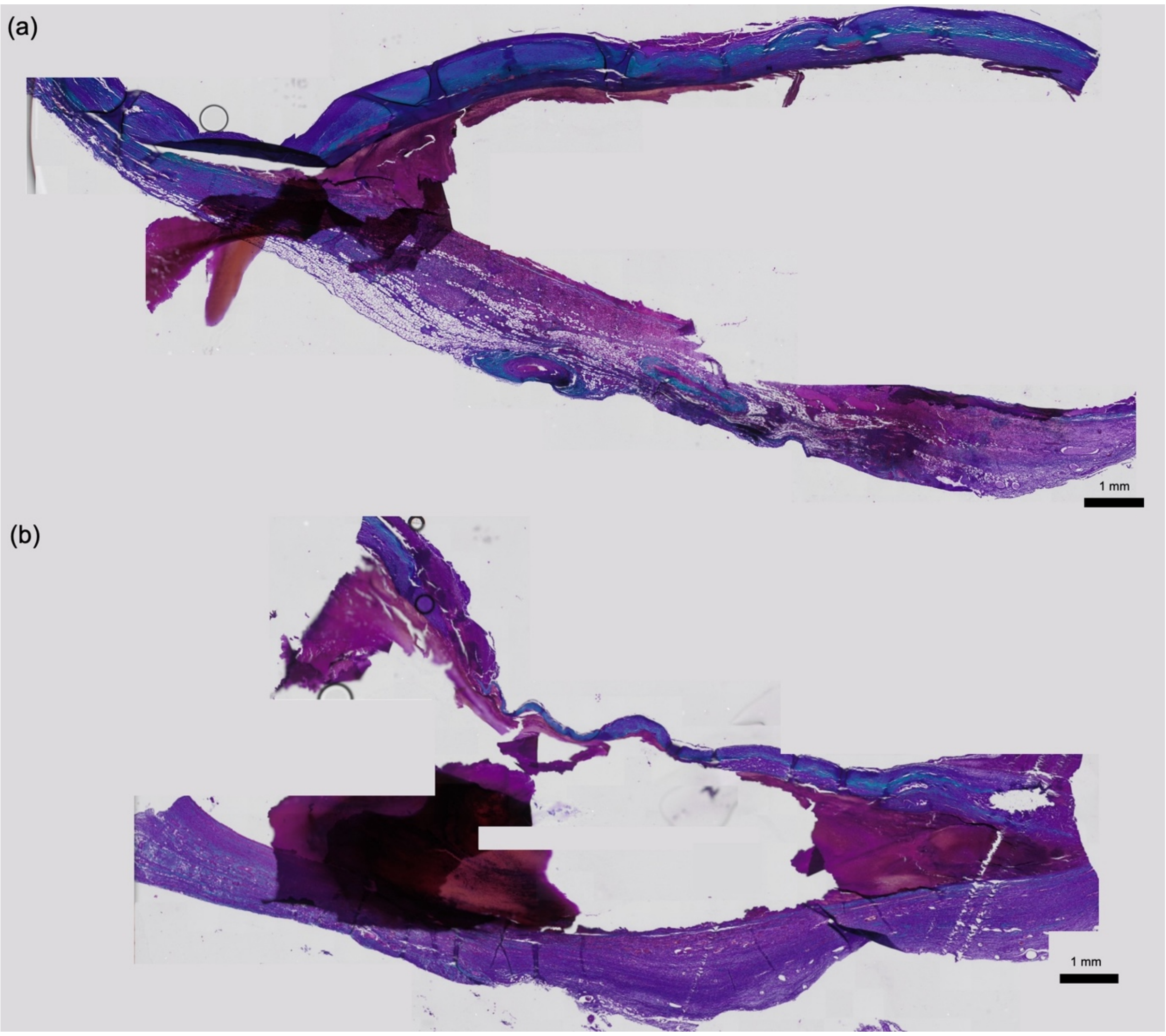

Supplementary figure 2. Additional example histological images of the TBAD specimen.

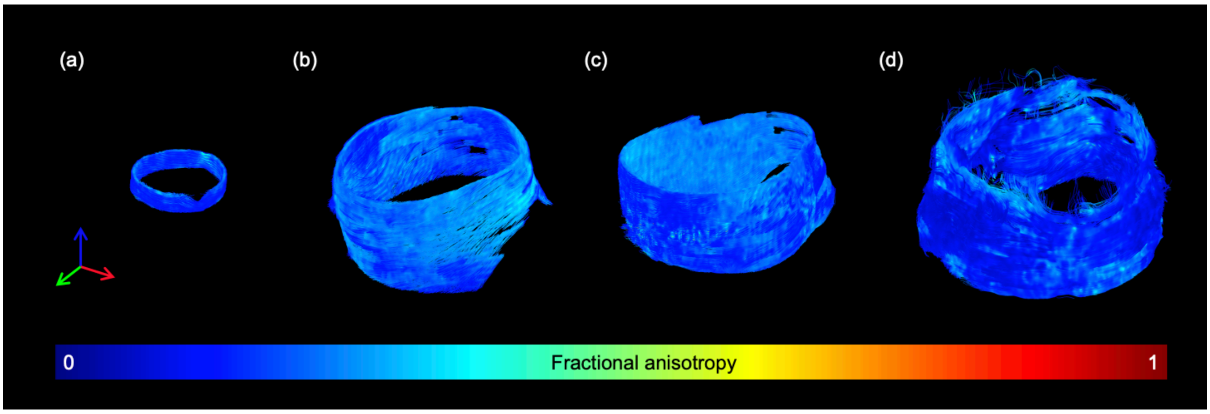

Supplementary figure 3. FA colour coded tractography of (a) healthy, (b, c) aneurysmal, and (d) TBAD aortic specimens.
